# Efficacy and Safety of Lenalidomide Monotherapy for Relapsed/Refractory Diffuse Large B Cell Lymphoma: Systematic Review and Meta-Analysis

**DOI:** 10.3389/fonc.2021.756728

**Published:** 2021-12-02

**Authors:** Jia Li, Jianpeng Zhou, Wei Guo, Xingtong Wang, Yangzhi Zhao, Ou Bai

**Affiliations:** ^1^ Department of Hematology, The First Hospital of Jilin University, Changchun, China; ^2^ Department of Hepatological Surgery, The First Hospital of Jilin University, Changchun, China

**Keywords:** diffuse large B-cell lymphoma, lenalidomide, monotherapy, treatment outcome, systematic review, meta-analysis

## Abstract

**Introduction:**

Several maintenance therapies are available for treatment of patients with relapsed/refractory (R/R) diffuse large B cell lymphoma (DLBCL). The objective of this review was to assess the efficacy and safety of lenalidomide monotherapy in these patients.

**Methods:**

MEDLINE, EMBASE, and the Cochrane Library databases were searched for publications up to April 7, 2021. Original studies that had information on lenalidomide monotherapy for DLBCL patients with R/R status were included. Meta-analyses of response rates, adverse events (AEs), overall survival (OS), and progression-free survival (PFS) were performed. The pooled event rates were calculated using a double arcsine transformation to stabilize the variances of the original proportions. Subgroup analysis was used to compare patients with different germinal center B-cell-like (GCB) phenotypes.

**Results:**

We included 11 publications that examined DLBCL patients with R/R status. These studies were published from 2008 to 2020. The cumulative objective response rate (ORR) for lenalidomide monotherapy was 0.33 (95% CI: 0.26, 0.40), and the ORR was better in patients with the non-GCB phenotype (0.50; 95% CI: 0.26, 0.74) than the GCB phenotype (0.06; 95% CI: 0.03, 0.11). The major serious treatment-related AEs were neutropenia, thrombocytopenia, respiratory disorders, anemia, and diarrhea. The median PFS ranged from 2.6 to 34 months and the median OS ranged from 7.8 to 37 months.

**Conclusion:**

This study provides evidence that lenalidomide monotherapy was active and tolerable in DLBCL patients with R/R status. Patients in the non-GCB subgroup had better responsiveness.

## Introduction

Diffuse large B cell lymphoma (DLBCL) is the most common subtype of non-Hodgkin lymphoma (NHL) and accounts for about 40% of all diagnosed lymphomas (1). The current standard first-line treatment of DLBCL is immunochemotherapy with rituximab plus cyclophosphamide, hydroxydaunorubicin, vincristine, and prednisolone, a regimen that provides complete and sustained remission for about 75% of newly diagnosed patients (2). The remaining patients are classified as having “relapsed” DLBCL if there is any new lesion after complete response (CR), and as “refractory” DLBCL if 50% or more of the lesions increased in size following initial treatment or if there is appearance of a new lesion during or following the initial treatment (3).

For DLBCL patients with relapsed/refractory (R/R) disease, the standard therapeutic option for those who are chemosensitive to second-line regimens is high-dose therapy plus autologous stem cell transplantation (ASCT) (4). Patients who are ineligible for ASCT or who fail after second-line treatment typically have poor prognoses. However, recent findings indicated that these patients may benefit from alternative salvage therapies. For example, lenalidomide with tafasitmab is often an effective treatment for DLBCL patients with R/R status.

Lenalidomide is a second−generation immunomodulatory drug, and several clinical trials reported that it provided effective treatment of multiple myeloma, myelodysplastic syndrome, and mantle cell lymphoma (5, 6). Other trials showed that lenalidomide monotherapy was an active and safe treatment for DLBCL patients with R/R status (7, 8). However, there has been no systematic synthesis of available studies on this topic.

The objective of the present study was to assess the efficacy and safety of lenalidomide monotherapy for DLBCL patients with R/R status and provide useful guidance for the treatment of these patients in clinical settings.

## Materials and Methods

### Search Strategy

**Figure 1:**
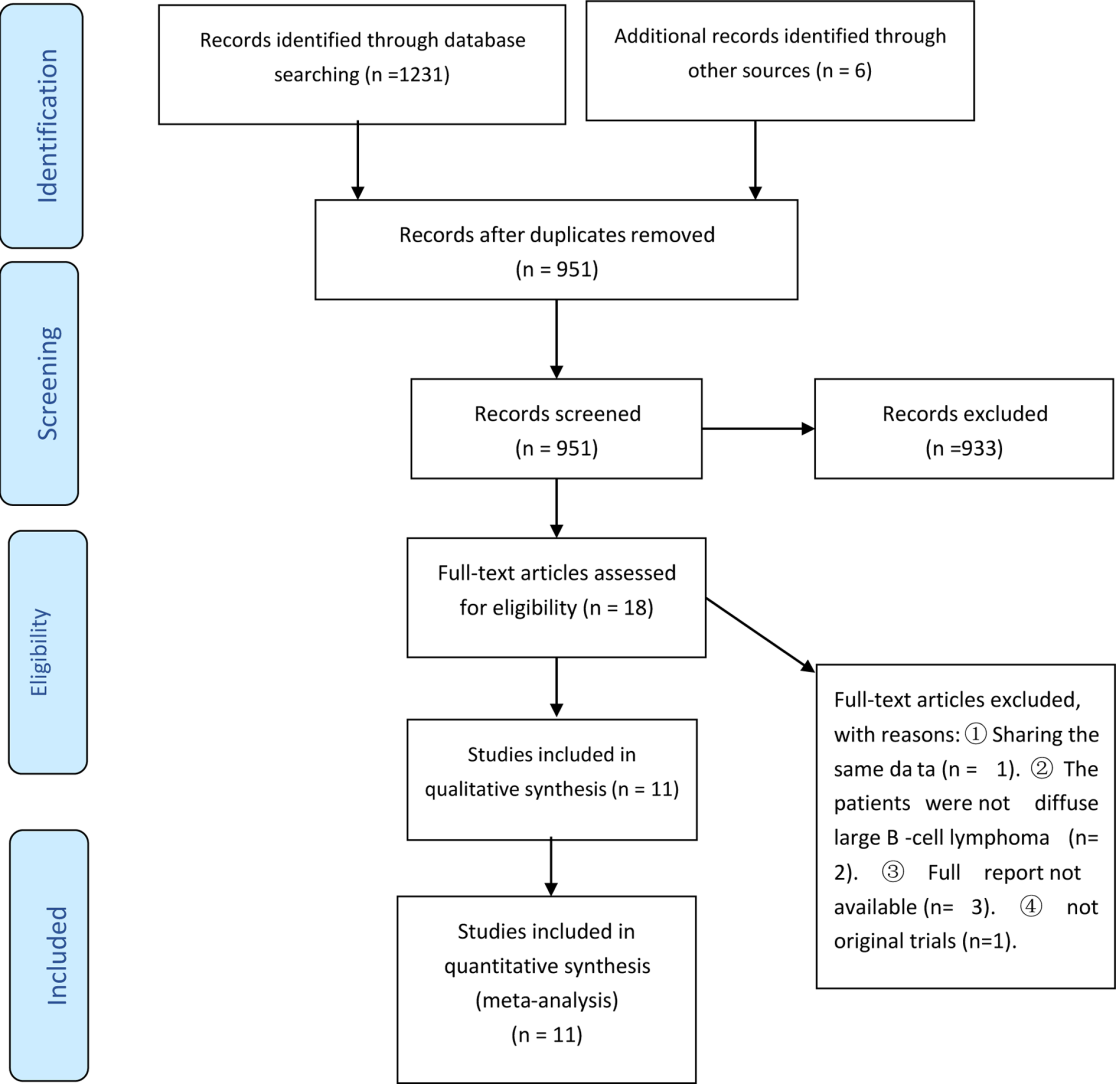
PRISMA flow diagram for selection of publications included in the meta-analysis.

The present systematic review and meta-analysis followed the PRISMA statement (9, 10) and used searches from Embase, Medline, and the Cochrane library to identify articles published up to April 7, 2021 (**Figure 1**). The search terms included “lenalidomide”, “diffuse large B-cell lymphoma”, and “lymphoma”, and appropriate search strategies and syntax were used for each database (**Appendix I**).

### Selection Criteria and Study Selection

The criteria for inclusion/exclusion were as follows: (*i*) studies were included if they were original randomized clinical trials, prospective cohort studies, prospective one-arm studies, or observational studies, but excluded if they were letters, commentaries, conference abstracts, case reports, case series, preclinical trials, review articles, or meta-analyses; (*ii*) studies were included if they examined populations of DLBCL patients with R/R status; (*iii*) studies were included if they provided information on lenalidomide monotherapy; and (*iv*) studies were included if they provided information on the outcomes of response rate, safety events, and survival [overall survival (OS) and progression-free survival (PFS)].

The titles and abstracts were first independently screened by two authors (Ou Bai and Jia Li) to identify potentially eligible publications. Then, full-text screening was independently performed by Wei Guo and Jia Li. Disagreements were resolved by discussion or by referral to a third party.

### Data Collection

Jia Li, Xingtong Wang, and Yangzhi Zhao performed the data collection independently and resolved disagreements by discussion or referral to a third party. The basic information of the included studies was study design; publication year; patient demographics; and data on response rates, safety events, and survival (OS and PFS). Responses were determined using the Cheson criteria, and included ORR, CR, partial response (PR), stable disease (SD), and progressive disease (PD) (3). PFS was defined as the time from the onset of lenalidomide monotherapy until PD (defined by RECIST criteria ver. 1.1) (11). OS time was defined as the time from the onset of lenalidomide monotherapy until death. Adverse events were reported and graded according to CTCAE ver. 5.0 (12).

### Data Analysis

Because the target was the efficacy and safety of the one-arm intervention, not a comparison of groups, the risk of bias assessment was performed using the Risk of Bias in Non-​randomized Studies of Interventions (ROBINS-I) tool (13). Meta-analyses of response rates, safety events, and survival rates (OS and PFS) were performed. Sensitivity analyses were not performed due to the limited amount of data. The pooled event rates were calculated using a double arcsine transformation to stabilize the variances of the original proportions. Each pooled rate is presented as proportion with a 95% confidential interval (CI). Heterogeneity was estimated using the Q-test. When the *P*-value was less than 0.1 (Q-test) and the I^2^ was greater than 50%, the result was considered heterogeneous, and a random-effects model was used for analysis; otherwise, a fixed-effects model was used. Subgroup analysis was performed to examine patients with germinal center B-cell-like (GCB) phenotype and non-GCB phenotype. A *P*-value below 0.05 was considered significant. All statistical analyses were performed using Stata version 15.0 (Stata Corp. Texas, USA).

## Results

### Basic Characteristics of Studies

Our initial screening led to the identification of 1237 potentially eligible studies (1231 from PubMed, EMBASE, and Cochrane Library, and 6 from other sources). We ultimately excluded 1226 of these studies based on the inclusion and exclusion criteria, and included 11 publications from 10 studies from that were published from 2008 to 2020 (**Figure 1** and **Table 1**) (7, 8, 14–22). Five of these studies were prospective one-arm studies (7, 8, 15, 16, 20, 21), four were retrospective analyses (14, 17, 18, 22), and one was a randomized controlled trial (19). The sample size ranged from 15 to 153 patients, and the median patient age ranged from 51 to 79 years old. Based on the ROBINS-I tool, the included studies had variable quality (**Table 2**). Moreover, because these data were from one-arm interventions, each study had a high risk of confounding. We also classified six studies as having problems with selection bias. The one RCT, in which our extracted data were targeted as a one-arm treatment, also had a high risk of confounding.

**Table 1:** Characteristics of included publications.

Wiernik et al. (8)						
Design		Single-arm, multicenter, open-label, phase II study in USA from August 2005 to September 2006				
Patient population		Relapsed/refractory aggressive NHL				
Overall sample		49 patients with relapsed/refractory aggressive NHL, 26 patients with DLBCL				
Age (years), median (range)		Whole cohort		Patients with DLBCL		
		65 (23, 86)		Not specified		
Male, n/N (%)		Whole cohort		Patients with DLBCL		
		25/49 (51.0)		Not specified		
Baseline characteristics	IPI score, n/N (%)			Whole cohort	Patients with DLBCL	
		0–1		8/49 (16.3)	Not specified	
		2–3		35/49 (71.4)	Not specified	
		4–5		6/49 (12.2)	Not specified	
	ECOG performance status, n/N (%)	Not specified.				
	ISS disease stage, n/N (%)	Not specified				
	Median number of prior treatment regimens	4				
	Patients with GCB	Not specified				
Maintenance therapy		Oral lenalidomide (25 mg once daily) on days 1 to 21 of every 28-day cycle. Patients continued therapy for 52 weeks as tolerated or until disease progression				
Outcomes		Response and safety				
**Hernandez-Ilizaliturri et al. (** 14 **)**						
Design		Retrospective one-arm study that reviewed data in USA for an unspecified period				
Patient population		Relapsed/refractory DLBCL				
Overall sample		40 overall, 23 with GCB, 17 with non-GCB				
Age (years), median (range)		Whole cohort	GCB	Non-GCB		
		66 (43, 80)	65 (46, 73)	68 (43-80)		
Male, n/N (%)		Whole cohort	GCB	Non-GCB		
		24/40 (60.0)	13/23 (56.5)	11/17 (64.7)		
Baseline characteristics	IPI score, n/N (%)		Whole cohort	GCB		Non-GCB
		0–1	11/40 (27.5)	8/23 (34.8)		3/17 (17.6)
		2–3	17/40 (42.5)	10/23 (43.5)		7/17 (41.2)
		4–5	12/40 (30.0)	5/23 (21.7)		7/17 (41.2)
	ECOG performance status, n/N (%)	Not specified.				
	ISS disease stage, n/N (%)		Whole cohort	GCB		Non-GCB
		I	4/40 (10.0)	3/23 (13.0)		1/17 (5.9)
		II	4/40 (10.0)	3/23 (13.0)		1/17 (5.9)
		III	12/40 (30.0)	8/23 (34.8)		4/17 (23.5)
		IV	20/40 (50.0)	9/23 (39.1)		11/17 (64.7)
	Median number of prior treatment regimens	Whole cohort	GCB	Non-GCB		
		4 (2, 13)	4 (2, 7)	4 (2, 13)		
	Patients with GCB, n/N (%)	23/40 (57.5)				
Maintenance therapy		All 40 patients in the final analysis received single-agent lenalidomide (25 mg once daily) for 21 days of a 28-day cycle. Patients continued lenalidomide until disease progression or unacceptable toxicity				
Outcomes		Response and survival outcomes				
**Witzig et al. (** 15 **)**						
Design		Single-arm, multicenter, open-label, phase II study in USA from November 2006 to March 2008				
Patient population		Relapsed/refractory aggressive NHL				
Overall sample		217 patients with relapsed/refractory aggressive NHL, and 108 patients with DLBCL				
Age (years), median (range)		Whole cohort		Patients with DLBCL		
		66 (21, 87)		Not specified.		
Male, n/N (%)		Whole cohort		Patients with DLBCL		
		140/217 (64.5)		Not specified.		
Baseline characteristics	IPI score, n/N (%)		Whole cohort	Patients with DLBCL		
		0–1	44/217 (20.3)	Not specified.		
		2–3	136/217 (62.7)	Not specified.		
		4–5	37/217 (17.1)	Not specified.		
	ECOG performance status, n/N (%)		Whole cohort	Patients with DLBCL		
		0	90/217 (41.5)	Not specified.		
		1	100/217 (46.1)	Not specified.		
		2	25/217 (11.5)	Not specified.		
		Missing	2/217 (0.9)	Not specified.		
	ISS disease stage, n/N (%)	Not specified.				
	Median number of prior treatment regimens (range)	3 (1, 13)				
	Patients with GCB, n/N (%)	Not specified.				
Maintenance therapy		Oral lenalidomide (25 mg once daily) on days 1 to 21 of every 28-day cycle until disease progression or unacceptable adverse events				
Outcomes		Response, safety, and survival				
**Lakshmaiah et al. (** 16 **)**						
Design		Prospective one-arm study in India from March 2011 to December 2012				
Patient population		Relapsed/refractory NHL				
Overall sample		25 patients with relapsed/refractory aggressive NHL, and 15 patients with DLBCL				
Age (years), median (range)		Whole cohort		Patients with DLBCL		
		51		Not specified.		
Male, n/N (%)		Whole cohort		Patients with DLBCL		
		140/217 (64.5)		Not specified.		
Baseline characteristics	IPI score, n/N (%)	Not specified.				
	ECOG performance status, n/N (%)	Not specified.				
	ISS disease stage, n/N (%)	Not specified.				
	Median number of prior treatment regimens	Not specified.				
	Patients with GCB, n/N (%)	Not specified.				
Maintenance therapy		Oral lenalidomide (starting at 20 mg/day and adjusted based on tolerability) from day 1 to 21 of every 28-day cycle until disease progression or unacceptable events				
Outcomes		Response, safety, and survival				
**Zinzani et al. (** 17 **)**						
Design		Retrospective one-arm study that reviewed data in Italy from April 2008 to November 2010				
Patient population		Relapsed/refractory aggressive NHL				
Overall sample		64 patients with relapsed/refractory aggressive NHL and 19 patients with DLBCL				
Age (years), median (range)		Whole cohort		Patients with DLBCL		
		71 (44, 84)		Not specified.		
Male, n/N (%)		Whole cohort		Patients with DLBCL		
		43/71 (67.2)		Not specified.		
Baseline characteristics	IPI score, n/N (%)	Not specified.				
	ECOG performance status, n/N (%)	Not specified.				
	ISS disease stage, n/N (%)	Not specified.				
	Median number of prior treatment regimens	3 (1, 17)				
	Patients with GCB, n/N (%)	Not specified.				
Maintenance therapy		Lenalidomide monotherapy with unspecified details.				
Outcomes		Response, safety, and survival				
**Mondello et al. (** 18 **)**						
Design		Retrospective one-arm study that reviewed data in Italy from January 2006 to January 2015				
Patient population		Relapsed/refractory DLBCL				
Overall sample		123 overall, 57 with GCB, 66 with non-GCB				
Age (years), median		Whole cohort	GCB	Non-GCB		
		64	Not specified.	Not specified.		
Male, n/N (%)		Whole cohort	GCB	Non-GCB		
		75/123 (61.0)	Not specified.	Not specified.		
Baseline characteristics	IPI score, n/N (%)		Whole cohort	GCB		Non-GCB
		0–1	6/123 (4.9)	Not specified.		Not specified.
		2–3	75/123 (61.0)	Not specified.		Not specified.
		4–5	42/123 (34.1)	Not specified.		Not specified.
	ECOG performance status, n/N (%)		Whole cohort	GCB		Non-GCB
		>1	21/123 (17)	Not specified.		Not specified.
	ISS disease stage, n/N (%)		Whole cohort	GCB		Non-GCB
		I	3/123 (2.4)	Not specified.		Not specified.
		II	19/123 (15.4)	Not specified.		Not specified.
		III	23/123 (18.7)	Not specified.		Not specified.
		IV	78/123 (63.4)	Not specified.		Not specified.
	Prior treatment regimens, median (range)	1 (1, 3)				
	Patients with GCB, n/N (%)	57/123 (46.3)				
Maintenance therapy		Oral lenalidomide (15 mg/day) for 24 patients (GCB: n = 13; non-GCB, n = 11); oral lenalidomide (25 mg/day) for 99 patients (GCB: n = 44; non-GCB: n = 55)				
Outcomes		Response and survival				
**Czuczman et al. (** 19 **)**						
Design		Phase II/III multicenter, randomized, open-label international study from 2 September 2010 to 5 April 2018 (DLC-001 trial)				
Patient population		Relapsed/refractory DLBCL				
Overall sample		51 overall, 23 with GCB, 28 with non-GCB				
Age (years), median (range)		Whole cohort	GCB	Non-GCB		
		69 (28, 84)	70 (37, 84)	68 (28, 78)		
Male, n/N (%)		Whole cohort	GCB	Non-GCB		
		30/51 (58.8)	13/23 (56.5)	17/28 (60.7)		
Baseline characteristics	IPI score, n/N (%)	Not specified.				
	ECOG performance status, n/N (%)		Whole cohort	GCB		Non-GCB
		0	18/51 (35.3)	6/23 (26.1)		12/28 (42.9)
		1	24/51 (47.1)	12/23 (52.2)		12/28 (42.9)
		2	7/51 (13.7)	4/23 (17.4)		3/28 (10.7)
	ISS disease stage, n/N (%)	Not specified.				
	Prior treatment regimens		Whole cohort	GCB		Non-GCB
		1	5/51 (9.8)	2/23 (8.7)		3/28 (10.7)
		2	21/51 (41.2)	7/23 (30.4)		14/28 (50.0)
		≥3	25/51 (49.0)	14/23 (60.9)		11/28 (39.3)
		ASCT	13/51 (25)	6/23 (26.1)		7/28 (25.0)
	Patients with GCB, n/N (%)	23/51 (45.1)				
Maintenance therapy		Oral daily lenalidomide (25 mg for creatinine clearance ≥ 60 mL/min; 10 mg for creatinine clearance ≥ 30 mL/min and < 60 mL/min) for day 1 to 21 in each 28-day cycle until progressive disease (PD), unacceptable toxicity, or voluntary withdrawal				
Outcomes		Response, safety, and survival				
**Ferreri et al. (** 20 **, ** 21 **)**						
Design		Open label, single-arm, multicenter phase II trial in Italy from 24 March 2009 to 22 December 2015				
Patient population		Relapsed/refractory DLBCL.				
Overall sample		46 overall, 20 with GCB, and 19 with non-GCB				
Age (years), median (range)		Whole cohort	GCB	Non-GCB		
		72 (34, 86)	Not specified.	Not specified.		
Male, n/N (%)		Whole cohort	GCB	Non-GCB		
		27/46 (58.7)	Not specified.	Not specified.		
Baseline characteristics	IPI score, n/N (%)		Whole cohort	GCB		Non-GCB
		0–1	8/46 (17.4)	Not specified.		Not specified.
		2–3	33/46 (71.7)	Not specified.		Not specified.
		4–5	5/46 (10.9)	Not specified.		Not specified.
	ECOG performance status, n/N (%)		Whole cohort	GCB		Non-GCB
		0	29/46 (63.0)	Not specified.		Not specified.
		1	15/46 (32.6)	Not specified.		Not specified.
		2	1/46 (2.2)	Not specified.		Not specified.
		3	1/46 (2.2)	Not specified.		Not specified.
	ISS disease stage, n/N (%)		Whole cohort	GCB		Non-GCB
		Advanced stage	35/46 (76.1)	Not specified.		Not specified.
	Prior treatment regimens, median (range)	Not specified				
	Patients with GCB, n/N (%)	20/39 (51.3)				
Maintenance therapy		Oral lenalidomide (25 mg per day for 21 days every 28 days) started within 2 months from salvage chemotherapy conclusion and until lymphoma progression or unacceptable toxicity (severely compromised organ function, quality of life, or both)				
Outcomes		Response, safety, and survival				
**Beylot-Barry et al. (** 7 **)**						
Design		Open-label, multicenter, single-arm, two-stage, phase II clinical trial in France from July 2012 to September 2014				
Patient population		Relapsed/refractory primary cutaneous DLBCL, leg type				
Overall sample		19				
Age (years), median (range)		79 (69, 92)				
Male, n/N (%)		3/19 (15.8)				
Baseline characteristics	IPI score, n/N (%)	Not specified				
	ECOG performance status, n/N (%)	0	12/19 (63.2)			
		1	5/19 (26.3)			
		2	2/19 (10.5)			
	ISS disease stage, n/N (%)	Not specified.				
	Median number of prior treatment regimens (range)	6 (1, 13)				
	Patients with GCB, n/N (%)	Not specified				
Maintenance therapy		Oral lenalidomide (25 mg once daily) on days 1 to 21 of every 28-day cycle for 12 cycles, as tolerated or until disease progression				
Outcomes		Response and safety				
**Broccoli et al. (** 22 **)**						
Design		Retrospective one-arm study that reviewed data in Italy from May 2011 to January 2015				
Patient population		Relapsed/refractory DLBCL				
Overall sample		153				
Age (years), median (range)		72 (25, 93)				
Male, n/N (%)		75/153 (49.0)				
Baseline characteristics	IPI score, n/N (%)	Not specified				
	ECOG performance status, n/N (%)	0–1	110/153 (71.9)			
		2	30/153 (19.6)			
		3	13/153 (8.5)			
	ISS disease stage, n/N (%)	I/II	37/153 (24.2)			
		III	35/153 (22.9)			
		IV	81/153 (52.9)			
	Median number of prior treatment regimens (range)	Not specified.				
	Patients with GCB, n/N (%)	Not specified.				
Maintenance therapy		Oral lenalidomide (starting dose of 10, 15, 20, 25 mg/day) for 21 days of a 28-day cycle until disease progression or relapse; initial dosing and dose adjustments at the physician’s discretion				
Outcomes		Response, safety, and outcome				

NHL, non-Hodgkin’s lymphoma; ECOG, Eastern Cooperative Oncology Group; DLBCL, diffuse large B-cell lymphoma; GCB germinal center B-cell–like; IPI, International Prognostic Index; ISS, International Staging System.

**Table 2:** Results from the risk of bias in non-​randomized studies of interventions (ROBIN-I) tool.

Author (year)	Confounding	Selection of participants	Classification of interventions	Deviations from intended interventions	Missing data	Measurement of outcomes	Selection of reported result	Risk of Bias score
**Wiernik et al.** (8)	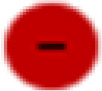	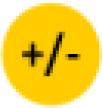				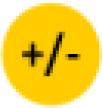		4/7
**Hernandez-Ilizaliturri et al.** (14)	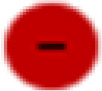	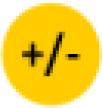			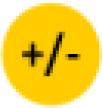			4/7
**Witzig et al.,** (15)	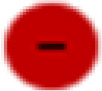							6/7
**Lakshmaiah et al.** (16)	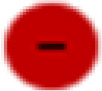	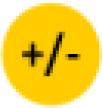				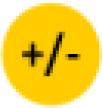		4/7
**Zinzani et al.** (17)	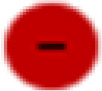	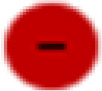			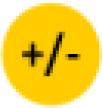			4/7
*Mondello et al. (18)	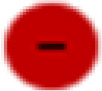	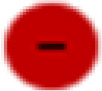						5/7
**Czuczman et al.** (19)	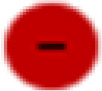							6/7
**Ferreri et al. 2017&2020** (20, 21)	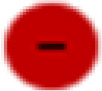							6/7
**Beylot-Barry et al.** (7)	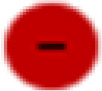							6/7
**Broccoli et al.** (22)	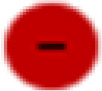	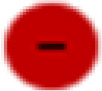			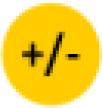			4/7


low bias, 
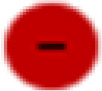
high bias, 
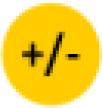
unclear bias.

*Randomized controlled trial that was only analyzed as a one-arm assessment observational study.

### Response Rates and Adverse Events

**Figure 2:**
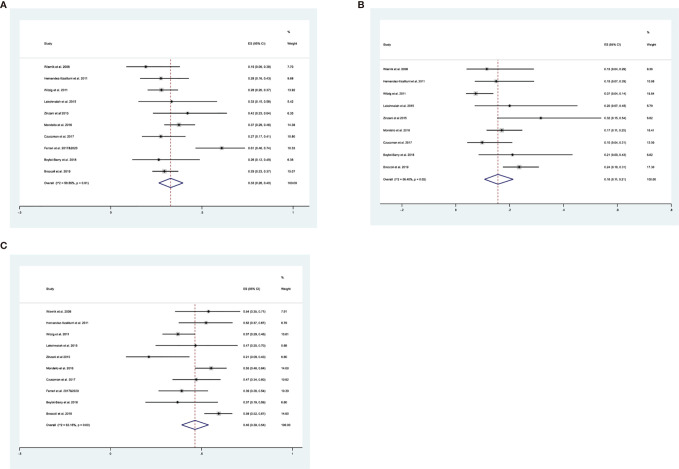
**(A)** Forest plot of the overall response rates of patients who received maintenance treatment consisting of lenalidomide monotherapy. **(B)** Forest plot of the complete response rates of patients who received maintenance treatment consisting of lenalidomide monotherapy. **(C)** Forest plot of progressive disease rates of patients who received maintenance treatment consisting of lenalidomide monotherapy.

All publications reported ORRs, and the pooled results had an ORR of 0.33 (95% CI: 0.26, 0.40, I^2^ = 59.55%; **Figure 2A**). Among all 600 patients, 197 achieved at least PR. The cumulative CR (which included confirmed and unconfirmed CR) was 0.16 (95% CI: 0.11, 0.21, I^2^ = 56.40%; **Figure 2B**). PD was present in about half the patients, and the cumulative PD was 0.46 (95% CI: 0.39, 0.54, I^2^ = 63.18%; **Figure 2C**). We also determined several other responses (**Table 3**). Notably, the median response duration ranged from 4.1 months to 18.5 months (**Table 4**).

**Table 3:** Pooled response rates and five major adverse events (≥Grade 3) in patients who received maintenance treatment consisting of lenalidomide monotherapy.

Efficacy		
**Response**	**Pooled response rate (95% CI)**	**Number of studies (patients)**
ORR	0.33 (0.26, 0.40)	10 (600)
CR/CRu	0.16 (0.11, 0.21)	9 (554)
PR	0.13 (0.08, 0.18)	9 (554)
SD	0.18 (0.12, 0.24)	9 (554)
PD	0.46 (0.39, 0.54)	10 (600)
**Safety**		
**Adverse events**	**Rate (95% CI)**	**Number of studies (patients)**
Neutropenia	0.28 (0.20, 0.37)	4 (269)
Thrombocytopenia	0.06 (0.01, 0.12)	4 (269)
Respiratory disorder	0.05 (0.03, 0.09)	2 (204)
Anemia	0.04 (0, 0.11)	4 (269)
Diarrhea	0.02 (0, 0.06)	3 (218)

ORR, objective response rate; CR, complete response; CRu, complete remission unconfirmed; PR, partial response; SD, stable disease; PD, progressive disease.

**Table 4:** Progression-free survival (PFS) and overall survival (OS) in patients who received maintenance treatment consisting of lenalidomide monotherapy.

Reference		Follow-up, median months (range)	PFS		OS		Response duration
			Median months (95% CI)	Mean % (95% CI)	Median, months (95% CI)	Median % (95% CI)	Median, months (95% CI), months
**Hernandez-Ilizaliturri et al. (** 14 **)**	**All**	Not specified	2.6 (0.9, 4.2)	Not specified	Not specified.	Not specified	Not specified.
	**GCB**		1.7 (0.3, 3.1)		13.5 (0, 33)		
	**Non-GCB**		6.2 (2.9, 9.6)		14 (7.3, 20.6)		
**Witzig et al. (** 15 **)**		9.2	2.7	Not specified	Not specified.	Not specified	4.6
**Zinzani et al. (** 17 **)**		Not specified	10.9 (1.2, not yet reached)	Not specified	Not specified.	Not specified	5.7
**Mondello et al. (** 18 **)**	**All**	54 (2, 108)	34 (2, 108)	Not specified	37 (7, 127)	Not specified	9 (1, 23)
	**GCB**		30 (2, 74)		41 (18, 68)		5 (1, 10)
	**Non-GCB**		37 (9, 108)		38 (7, 127)		15 (5, 23)
**Czuczman et al. (** 19 **)**	**All**	Not specified	3.4	Not specified	7.8	Not specified	18.5 (4.1, not yet reached)
	**GCB**	2.5	7.5				
	**Non-GCB**	3.8	8.1				
**Ferreri et al. (** 20, 21 **)**	**All**	Not specified	Not specified	1 yr: 70% (57, 83); 5 yrs: 48% (41, 55).	Not specified	1 yr: 81% (70, 92); 3 yrs: 71% (57, 85); 5 yrs: 62% (55, 69).	Not specified
	**GCB**			1 yr: 64% (44, 84)		Not specified	
	**Non-GCB**			1 yr: 67% (47, 87)		Not specified	
**Beylot-Barry et al. (** 7 **)**		49 (20, 52)	4.9	Not specified	19.4	Not specified	4.1
**Broccoli et al. (** 22 **)**		36	6	14.6% at 80 months	12	27.7% at 80 months	Not specified.

GCB, germinal center B-cell-like.

**Figure 3:**
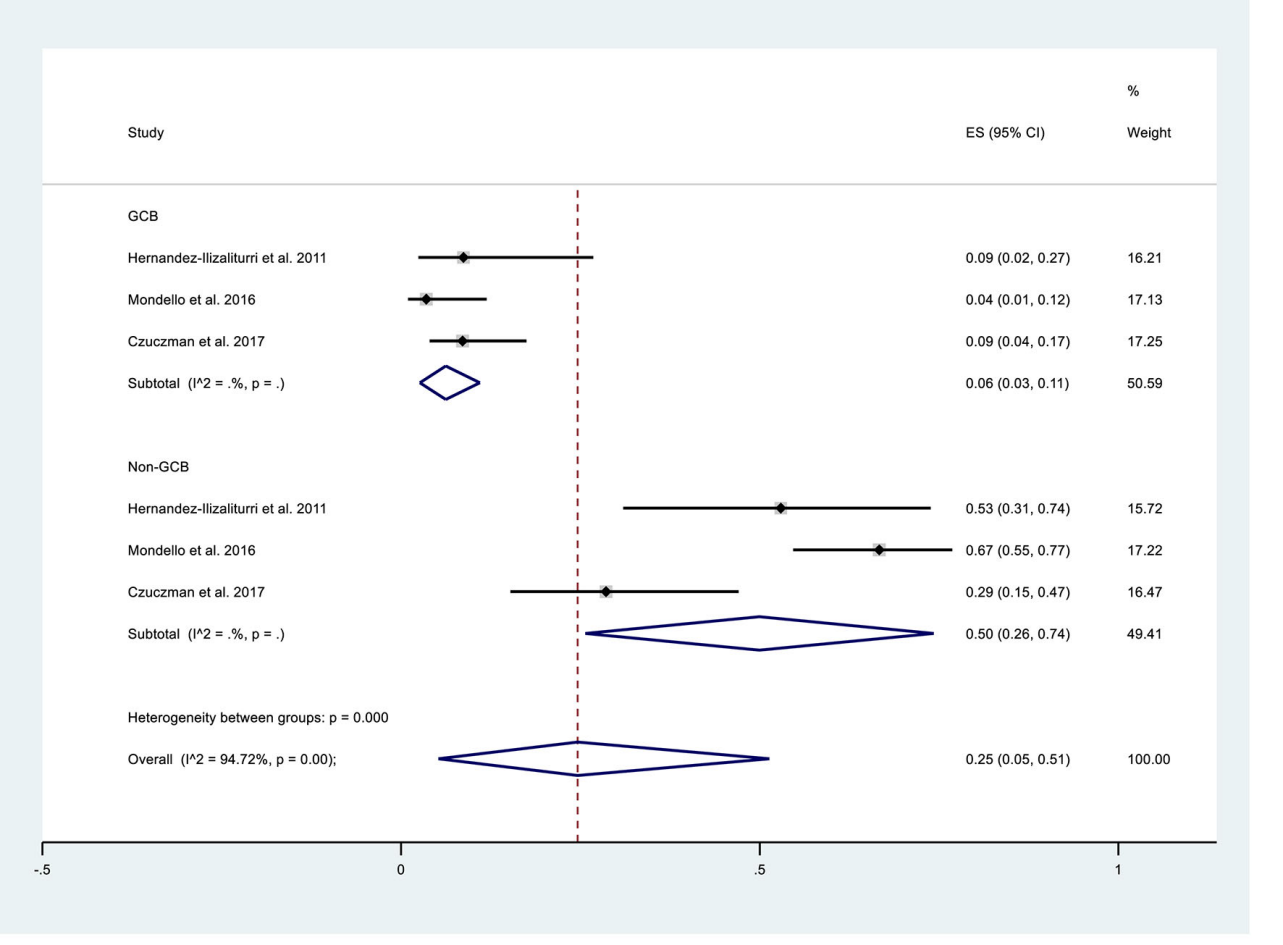
Subgroup analysis of overall response rates of patients with germinal center B-cell-like (GCB) phenotype or non-GCB phenotype who received maintenance treatment consisting of lenalidomide monotherapy.

We performed subgroup analysis to compare the responses of patients with the GCB and non-GCB phenotypes (**Table 5**, **Figure 3**). The results indicated that patients with non-GCB status had a greater ORR (0.50; 95% CI: 0.26, 0.74) than those with GCB status (0.06; 95% CI: 0.03, 0.11). The non-GCB group also had significantly better CR and PR (both *P* < 0.05).

**Table 5:** Pooled response rates in patients with GCB and non-GCB phenotypes who received maintenance treatment consisting of lenalidomide monotherapy.

Response	GCB (3 studies, 150 patients)	Non-GCB (3 studies, 111 patients)
ORR (95% CI)	*0.06 (0.03, 0.11)	0.50 (0.26, 0.74)
CR/CRu (95% CI)	*0.01 (0, 0.03)	*0.26 (0.18, 0.35)
PR (95% CI)	*0.05 (0.02, 0.09)	*0.26 (0.18, 0.35)
SD (95% CI)	0.12 (0.03, 0.25)	0.10 (0, 0.28)
PD (95% CI)	0.57 (0.09, 0.97)	0.32 (0.23, 0.41)

*Fixed-effects model.

GCB, germinal center B-cell-like; ORR, objective response rate; CR, complete response; Cru, complete remission unconfirmed; PR, partial response; SD, stable disease; PD, progressive disease.

The most serious treatment-related adverse events (AEs; Grade 3 or more) were neutropenia, thrombocytopenia, respiratory disorder, anemia, and diarrhea, and their mean cumulative incidences ranged from 2% to 28% (**Table 3**).

### Survival Data

Eight studies reported survival data. The median PFS ranged from 2.6 to 34 months and the median OS ranged from 7.8 to 37 months (**Table 4**). The study by Mondello et al. (18) reported distinctly better survival rates than the other studies. Further analysis indicated the Mondello et al. study examined patients who were less likely to be high-risk (34%), received fewer early treatment lines (mean: 1), and had longer median response times to the first treatment (median: 23 months).

### Publication Bias

**Figure 4:**
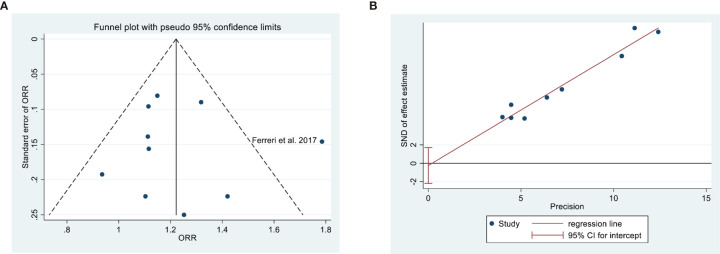
Assessment of publication bias in overall response rate based on a funnel plot **(A)** and Egger’s test (**B**, P = 0.778).

Analysis of publication bias indicated no evidence of this bias based on a symmetric funnel plot and the results of the Egger’s test (*P* = 0.778; **Figure 4**).

## Discussion

Our meta-analysis of 10 studies that examined the effect of lenalidomide monotherapy for DLBCL patients with R/R status indicated the ORR was 0.33 (95% CI: 0.26, 0.40). Moreover, patients with the non-GCB phenotype had a greater ORR (0.50; 95% CI: 0.26-0.74) than those with the GCB phenotype (0.06; 95% CI: 0.03, 0.11). The major serious treatment-related AEs in these patients were neutropenia, thrombocytopenia, respiratory disorder, anemia, and diarrhea. The median PFS ranged from 2.6 to 34 months and the median OS ranged from 7.8 to 37 months.

The introduction of lenalidomide treatment for DLBCL patients who have R/R status provides an opportunity for them to overcome chemorefractoriness (5). The anti-cancer effects of lenalidomide are due to its stimulation of cereblon, a component of E3 ubiquitin-ligase, and restoration of the function of immune effector cells (23). Our meta-analysis indicated the cumulative ORR (0.33; 95% CI 0.26, 0.40) was similar to that achieved by obinutuzumab monotherapy (0.32) (24) and tafasitamab monotherapy (ORR: 0.26–0.29) (25). Furthermore, trials have shown that combining lenalidomide and tafasitamab had higher efficacy than the single drug each, which indicated the synergistic effect between the two drugs (26, 27). Because lenalidomide is an immunomodulatory agent, clinicians have used it for maintenance therapy and in various induction and salvage regimens (28). However, the evidence of a benefit of lenalidomide for DLBCL patients with R/R status is still limited. Some trials (e.g., NCT03730740) are now examining the efficiency of lenalidomide monotherapy as maintenance treatment for R/R non-Hodgkin T-cell lymphoma.

The GCB and non-GCB phenotypes of DLBCL have significant differences in prognosis (29, 30), and these phenotype have approximately the same prevalence among DLBCL patients (31). Although there are several moderating factors, patients with the non-GCB phenotype have better prognosis (32). In agreement, our meta-analysis indicated the ORR, CR, and PR of the non-GCB subgroup were significantly better (all *P* < 0.05). This may be related to the effect of lenalidomide on the transcription regulatory factor IRF4/MUM1 and its inhibition of the nuclear factor-kB pathway (33, 34). Further large-scale trials are needed to confirm these findings.

Previous studies reported the AEs of lenalidomide monotherapy were generally manageable (5). The most frequent serious AE in our 10 included studies was neutropenia (0.28; 95% CI: 0.20, 0.37). One study that compared placebo with lenalidomide reported a greater risk of neutropenia in the lenalidomide group (RR: 4.74; 95% CI: 2.96, 7.57) (35). Therefore, in routine clinical practice, prevention and appropriate management of neutropenia are important when administering lenalidomide monotherapy.

Because of the limited data in the available studies, we were unable to assess survival rates. However, Mondello et al. reported better survival rates than the other studies due to their methods of patient selection. In particular, they included fewer patients with high-risk (34%), patients who received fewer early treatment lines (mean: 1), and patients who had longer median response times for the first treatment (median: 23 months) (18). Further investigations are needed to confirm the effects of these different factors on survival of these patients.

To our best knowledge, the present systematic review is the first to examine the effect of lenalidomide monotherapy for DLBCL patients with R/R status. Our results indicated this treatment was active and tolerable, but these results should be considered with caution because the data were mostly from low-quality observational studies. For instance, one of the limitations of the present systematic review is the presence of selection bias regarding patient inclusion. Large and rigorously designed studies on this topic are needed to confirm the efficiency and safety of lenalidomide monotherapy for DLBCL patients with R/R status.

## Conclusion

The results of the present study suggest that lenalidomide monotherapy was active for DLBCL patients with R/R status and leads to AEs that are mostly manageable. The non-GCB subgroup of these patients had greater tumor responsiveness than the GCB subgroup.

## Data Availability Statement

The raw data supporting the conclusions of this article will be made available by the authors, without undue reservation.

## Author Contributions

OB designed and JL performed most of the investigation, data analysis and wrote the manuscript. JZ, WG, XW, and YZ provided data collection assistance. JZ contributed to interpretation of the data and analyses. All authors contributed to the article and approved the submitted version.

## Conflict of Interest

The authors declare that the research was conducted in the absence of any commercial or financial relationships that could be construed as a potential conflict of interest.

## Publisher’s Note

All claims expressed in this article are solely those of the authors and do not necessarily represent those of their affiliated organizations, or those of the publisher, the editors and the reviewers. Any product that may be evaluated in this article, or claim that may be made by its manufacturer, is not guaranteed or endorsed by the publisher.
