# Supplementary material for: Efficacy and Safety of Lenalidomide Monotherapy for Relapsed/Refractory Diffuse Large B Cell Lymphoma: Systematic Review and Meta-Analysis
Source: Front Oncol. 2021 Dec 2;11:756728. doi: 10.3389/fonc.2021.756728 (PMC8674688; doi:10.3389/fonc.2021.756728)
Supplement: Supplementary file 1 [file Presentation_1.pdf]

Appendix I. Search strategies used for the different databases.

Cochrane Library

Date Run: 07/04/2021 19:55:49

ID	Search	Hits
#1	MeSH descriptor: [Lymphoma, Large B-Cell, Diffuse] explode all trees	420
#2	MeSH descriptor: [Plasmablastic Lymphoma] explode all trees	15
#3	(histiocytic next lymphoma*):ti,ab,kw	37
#4	(Diffuse Large-Cell next lymphoma*):ti,ab,kw	309
#5	(Diffuse Large B Cell Lymphoma*):ti,ab,kw	1508
#6	#1 or #2 or #3 or #4 or #5	1569
#7	MeSH descriptor: [Lenalidomide] explode all trees	405
#8	("lenalidomide"):ti,ab,kw	1992
#9	#7 or #8	1992
#10	#6 and #9	104

OVID Medline Epub Ahead of Print, In-Process & Other Non-Indexed Citations, Ovid MEDLINE(R) Daily and Ovid MEDLINE(R) 1946 to Present

1 exp Lymphoma, Large B-Cell, Diffuse/ (20341)

2 exp Plasmablastic Lymphoma/ (155)

3 (diffuse adj6 large* adj6 lymphoma*).mp. [mp=title, abstract, original title, name of substance word, subject heading word, floating sub-heading word, keyword heading word, organism supplementary concept word, protocol supplementary concept word, rare disease supplementary concept word, unique identifier, synonyms] (28028)

4 (histiocytic adj lymphoma*).mp. (1168)

5 1 or 2 or 3 or 4 (28729)

6 exp Lenalidomide/ (2830)

7 Lenalidomide.mp. (5010)

8 6 or 7 (5010)

9 5 and 8 (181)

Embase <1974 to 2021 April 06>

1 exp Lymphoma, Large B-Cell, Diffuse/ (15087)

2 exp Plasmablastic Lymphoma/ (1077)

3 (diffuse adj6 large* adj6 lymphoma*).mp. [mp=title, abstract, heading word, drug trade name, original title, device manufacturer, drug manufacturer, device trade name, keyword, floating subheading word, candidate term word] (33940)

4 (histiocytic adj lymphoma*).mp. (2717)

5 1 or 2 or 3 or 4 (37494)

6 exp Lenalidomide/ (20543)

7 Lenalidomide.mp. (21393)

8 6 or 7 (21393)

9 5 and 8 (946)
